# Foxp3^+^ Regulatory T Cells in Bone and Hematopoietic Homeostasis

**DOI:** 10.3389/fendo.2019.00578

**Published:** 2019-09-10

**Authors:** Luise Fischer, Caroline Herkner, Reni Kitte, Sebastian Dohnke, Julia Riewaldt, Karsten Kretschmer, Annette I. Garbe

**Affiliations:** ^1^Osteoimmunology, DFG-Center for Regenerative Therapies Dresden (CRTD), Technische Universität Dresden, Dresden, Germany; ^2^Molecular and Cellular Immunology/Immune Regulation, DFG-Center for Regenerative Therapies Dresden (CRTD), Technische Universität Dresden, Dresden, Germany

**Keywords:** bone disorders, bone microenvironment, lympho-hematopoiesis, osteoclasts, Foxp3^+^ Treg cells

## Abstract

The bone represents surprisingly dynamic structures that are subject to constant remodeling by the concerted action of bone-forming osteoblasts and bone-resorbing osteoclasts - two cell subsets of distinct developmental origin that are key in maintaining skeletal integrity throughout life. In general, abnormal bone remodeling due to dysregulated bone resorption and formation is an early event in the manifestation of various human bone diseases, such as osteopetrosis/osteoporosis and arthritis. But bone remodeling is also closely interrelated with lympho-hematopoietic homeostasis, as the bone marrow niche is formed by solid and trabecular bone structures that provide a framework for the long-term maintenance and differentiation of HSCs (>blood lineage cells and osteoclasts) and MSCs (>osteoblasts). Numerous studies in mice and humans have implicated innate and adaptive immune cells in the dynamic regulation of bone homeostasis, but despite considerable clinical relevance, the exact mechanisms of such immuno-bone interplay have remained incompletely understood. This holds particularly true for CD4^+^ regulatory T (Treg) cells expressing the lineage specification factor Foxp3: Foxp3^+^ Treg cells have been shown to play an indispensable role in maintaining immune homeostasis, but may also exert critical non-immune functions, which includes the control of metabolic and regenerative processes, as well as the differentiation of HSCs and function of osteoclasts. Here, we summarize our current knowledge on the T cell/bone interplay, with a particular emphasis on our own efforts to dissect the role of Foxp3^+^ Treg cells in bone and hematopoietic homeostasis, employing experimental settings of gain- and loss-of-Treg cell function. These data make a strong case that Foxp3^+^ Treg cells impinge on lympho-hematopoiesis through indirect mechanisms, i.e., by acting on osteoclast development and function, which translates into changes in niche size. Furthermore, we propose that, besides disorders that involve inflammatory bone loss, the modulation of Foxp3^+^ Treg cell function *in vivo* may represent a suitable approach to reinstate bone homeostasis in non-autoimmune settings of aberrant bone remodeling.

## Introduction

In adult mammals, the bone marrow (BM) is the primary site of hematopoiesis. Hematopoietic stem cells (HSCs) reside within specific niches, which are specialized microenvironments within the BM cavity, providing HSCs with regulatory signals essential for their maintenance, proliferation and differentiation into blood and immune cells. HSCs dynamically regulate their numbers by undergoing asymmetric, self-renewing divisions depending upon cell-intrinsic and cell-extrinsic mechanisms, thereby sustaining hematopoiesis over lifetime (1, 2). Homeostasis of the bone microenvironment critically depends on bone remodeling, a tightly regulated process involving three major cell types: bone-resorbing osteoclasts, bone-forming osteoblasts and osteocytes. Osteocytes originate from mature osteoblasts, residing within the lacuna of the mineralized bone matrix, and act as orchestrators of bone remodeling (3). Under physiological conditions, resorption of damaged bone by osteoclasts is followed by the recruitment of osteoblasts and the formation of new bone, a concerted action critical for the maintenance of skeletal integrity and bone homeostasis. Defects in osteoclastic activity cause osteopetrosis, a disease associated with high bone mass, whereas enhanced osteoclastic bone resorption results in low bone mass and osteoporosis (4). While the direct impact of osteoclasts on maintenance and BM retention of HSCs remains controversial (5–7), there is accumulating evidence that osteoblast lineage cells play a crucial role in the regulation of hematopoiesis (8–10). Since the majority of the cells in the BM are hematopoietic cells, it appears likely that, in addition to stromal niche components, HSCs are regulated by their own progeny. In this context, megakaryocytes have recently been identified as direct regulators of homeostatic HSC quiescence using several mechanisms, including secretion of CXCL4 and TGF-β (11–13), while direct cell-cell interaction of megakaryocytes and bone cells have been described to affect skeletal homeostasis (14–16). Similarly, it has been suggested that the function of osteoblasts in maintaining the HSC niche is controlled by macrophages, whereas “HSC niche macrophages” have been described to govern maintenance of hematopoiesis directly (17–20). However, due to the heterogeneity of macrophages, and since the bone- and BM-residing macrophage populations, including osteoclasts, are primarily defined by their histological location, the precise identity and role of the different macrophage subpopulations in bone remodeling, regeneration, and shaping the HSC niche still need to be defined (21–23).

The impact of cells of the adaptive immune system — B and T lymphocytes — on bone remodeling under physiological conditions is still a matter of debate: while some studies using lymphocyte-deficient mouse models such as *Rag*^−/−^, μMT or nude mice described low bone mass phenotypes, others did not (24–27). Given the fact that several populations of mature lymphocytes, such as long-lived memory T and B cells reside in the BM (28, 29) and share common niches with HSCs, it is reasonable to assume that these mature cells impinge on bone and hematopoietic homeostasis. In this context, it has been shown, that after allogeneic BM stem cell transplantation, T cells act as a double-edged sword as they can on the one hand promote graft-versus-host-disease (GvHD), but on the other hand can also provide beneficial effects on the engraftment (30). High-resolution imaging over time in an experimental setup of allogeneic HSC transplantation provided evidence that HSCs preferentially co-localize with CD4^+^ regulatory T (Treg) cells, expressing the lineage specification factor Foxp3, that accumulate on the endosteal surface of the bones of non-irradiated recipient mice, enabling transplanted stem cells to evade from allogeneic rejection (31, 32). The essential role of Foxp3^+^ Treg cells in maintaining immune homeostasis is firmly established (33, 34), but additional, non-immunological functions of Treg cells, such as controlling (i) the metabolic function in adipose tissue, (ii) regeneration of muscle cells, (iii) lympho-hematopoiesis, and (iv) osteoclast and osteoblast development and function, become increasingly apparent (35–41).

While much less is known about the interplay between Treg cells and osteoclasts/osteoblasts in bone remodeling and the formation of the HSC niche, a large body of experimental evidence focused on the immuno-bone crosstalk in rheumatoid arthritis (RA), one of the most common human autoimmune diseases. Osteoclasts play a central role in the pathogenesis of RA, where bone destruction is characterized by inflammatory osteolysis, caused by aberrant activation of the immune system, eventually leading to the destruction of surface cartilage and subchondral bone (42, 43). It has been proposed that the onset and pathogenesis of RA critically depends on the balance between two distinct CD4^+^ T cell subsets: type 17 helper T (TH17) cells, a pathogenic subset of CD4^+^ T cells that produces IL-17, and thereby promoting osteoclastogenesis, and Treg cells, crucial for the prevention of autoimmune diseases driven by TH17 cells (34, 44). The role of Treg cells in animal models for autoimmune arthritis has been demonstrated by amelioration of the disease following adoptive transfer of Treg cells and induction of an accelerated and more severe form of arthritis after depletion of Treg cells (45–48). Moreover, it has been described that under certain conditions of experimental arthritis a subpopulation of Foxp3^+^ Treg cells can convert into autoreactive Foxp3^−^ TH17 cells (49).

There is also evidence that Treg cells interact with osteoblasts. For example, studies on intermittent PTH-induced bone anabolism propose that Treg cells are involved in the up-regulation of the expression of the osteogenic factor Wnt10b by CD8^+^ T cells, which was also suggested to be the main mechanism in the promotion of bone formation by oral supplementation with the probiotic *Lactobacillus rhamnosus* GG (50–53). On the other hand, Treg cells have been implicated to play a role in bone formation by promoting the differentiation of osteoblasts directly (54).

Although the close relationship between the bone and the immune system has long been recognized (55), the spatial relationship and the interaction between the different cell types within the bone microenvironment as well as the identity of their communication factors, in particular under physiological conditions, is still incompletely understood.

Studies on the interplay between osteoclasts/osteoblasts and Treg cells in the BM microenvironment are hampered by several unresolved issues: (a) osteoclasts are difficult to study due to the lack of reliable methods for their *ex vivo* purification, owing to their low abundance, large size, and lack of specific surface marker expression. Furthermore, the phenotypic definition of “true” osteoclast precursors and their developmental stages vary considerably; (b) constitutive Treg cell deficiency inevitably results in secondary effects due to systemic autoimmunity and increased systemic levels of inflammatory factors. Mice with constitutive Treg cell deficiency suffer from severe morbidity leading to premature death prior to completion of bone development; (c) due to the unique properties and structure of bone, it is technically challenging to assess and visualize interactions between cells in the BM niche. Thus, it will be essential to develop experimental systems and more advanced imaging that keep these limitations to a minimum.

In this review we discuss the impact of BM-residing Treg cells on the bone microenvironment, central to the development of therapeutic strategies for the treatment of bone diseases and to promote tolerance after stem cell transplantation.

## Lympho-Hematopoietic Niche and Foxp3^+^ Treg Cells

For a long time, HSCs were considered as dormant cells but increasing evidence suggests HSCs as direct targets of inflammatory signals. Earlier studies have identified HSCs as “first responders” during inflammatory responses, e.g., during infections, later it became clear that pro-inflammatory cytokines such as interleukin (IL)-1, IL-6, IL-8, tumor necrosis factor (TNF) and type I and type II interferons (IFNs), G-CSF, and Toll-like receptor (TLR) ligands regulate HSCs not only in response to stress but also under homeostatic conditions. Together with BM niche signals such as CXCL12, basal levels of inflammatory cytokines provided by T cells, NK cells, neutrophils and macrophages control the balance between HSC dormancy and lineage fate decision under homeostatic conditions, while inflammatory conditions promote HSC proliferation and differentiation at the expense of self-renewal, emphasizing the interdependency of the distinct BM niche components in health and disease (56–60). However, increasing evidence is pointing towards regulation of HSC maintenance by distal/systemic factors: in addition to the nervous system (e.g., by oscillation of CXCL12 production) and hormones such as PTH or estrogen that have been described to regulate HSCs from the outside, two recent studies demonstrate that also the liver and the intestine contribute to HSC maintenance under steady-state conditions (61–65). Given that bone remodeling is also highly regulated by systemic factors, further studies are required to dissect direct and indirect contributions of distal organs on hematopoietic and skeletal homeostasis.

In both mouse and man, the T cell compartment in the BM, which constitutes only about 5% of mononuclear BM cells, is characterized by a lower CD4/CD8 T cell ratio and notably, by substantially elevated frequencies of Foxp3^+^ Treg cells within the CD4^+^ T cell population compared to peripheral lymphoid organs (66, 67). Like other BM T cells, BM Treg cells exhibit a more activated/memory phenotype. Transcriptional characterization of BM Treg cells revealed a signature distinct from Treg cells in the periphery. The differential expression of cytokine/chemokine receptors such as *Il9r, Ccr2*, and *Cxcr3* and effector molecules such as *Il10* and *Ctla4* suggests increased suppressive capacity of BM resident Treg cells (68). The transcriptional signature is consistent with the idea that these niche-associated Treg cells contribute to the maintenance of the BM microenvironment as an immune privilege site, crucial for HSC survival (31, 32).

**Figure 1:**
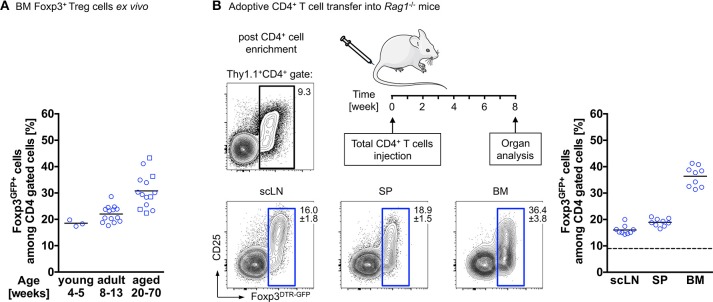
Accumulation of Foxp3^+^ Treg cells in the BM. **(A)** Age-related accumulation of BM-resident Foxp3^+^ Treg cells. Flow cytometry analysis of Foxp3^GFP+^ cells within the CD4^+^ T cell population in the BM of wildtype mice of different age groups. Each experiment was performed with at least 3 mice and symbols and lines indicate individual mice and mean values, respectively (young: 4–5 weeks; adult: 8–13 weeks; aged: circles: 20–22 weeks; and squares: 50–70 weeks). **(B)** Schematic overview of the experimental design. Four-week-old *Rag1*^−/−^ mice were adoptively transferred with bulk CD4^+^ T cells (upper plot: Foxp3^+^ Treg cell proportion among total CD4^+^ T cells before adoptively transferred). The distributions of gated Foxp3^DTR−GFP+^ cells in various organs of the recipient mice (scLN, subcutaneous lymph nodes; SP, spleen, and BM, bone marrow) were analyzed by flow cytometry 8 weeks after transfer. Numbers in plots indicate the mean percentages ± SD of gated cells within the respective gate. Graph illustrates the accumulation of Foxp3^+^ Treg cells in the BM. Data are collected from two independent experiments with 4–5 mice; symbols and lines denote individual mice and mean values, respectively.

Interestingly, and reminiscent of the above-described observations, our temporal analysis of Treg cells in the BM of wildtype mice revealed that the proportion of Foxp3^+^ Treg cells among CD4^+^ T cells further increases with age. While in juvenile mice, Foxp3^+^ Treg cells account for approximately 20% of the CD4^+^ T cell population, their frequency has almost doubled in aged mice (Figure 1A). This can be recapitulated by adoptive Treg cell transfers into Treg cell-deficient mice: here we found Foxp3^GFP+^ Treg cells, which initially comprised about 10–15% of the adoptively transferred splenic bulk CD4^+^ T cell population, to be selectively enriched in the BM of Foxp3-deficient recipient mice [up to 90% of transferred CD4^+^ T cells (69)] and in the BM of T and B cell-deficient *Rag1*^−/−^ recipient mice (up to 40% of transferred CD4^+^ T cells; Figure 1B), indicating the physiological relevance of the enrichment. Overall, these observations raise the question whether this accumulation of Foxp3^+^ Treg cells within the BM is due to preferential *de novo* induction of initially Foxp3-negative CD4^+^ T cells or preferential recruitment of preformed cells to the bone microenvironment (70, 71).

Since CD4^+^ Treg cells were originally identified exclusively by the constitutive expression of the IL-2 receptor alpha chain CD25, employing depleting antibodies directed against CD25 still represents the most widely used loss-of-function approach to characterize Treg cell function *in vivo* (72, 73). Whether the *in vivo* administration of anti-CD25 monoclonal antibodies (mAbs) leads to depletion or to functional inactivation of CD25-expressing Treg cells has been controversially discussed (74–77). In mouse models for RA, administration of anti-CD25 mAbs exacerbates the disease (45, 78, 79). However, interpretation of results from studies using this approach to assess the direct impact of Treg cells on bone cells is hampered due to the upregulation of CD25 expression on activated CD4 and CD8 T cells and in addition, due to the existence of CD25-negative Treg cells in particular in peripheral tissues and the BM. Consistently, anti-CD25 treatment spares Foxp3^+^ Treg cells with a CD25^low/−^ phenotype (80).

Only the identification of Foxp3 as the Treg cell lineage specification factor, necessary and sufficient for their development and function (81–83) allowed specific *in vivo* targeting of Foxp3^+^ Treg cells and with this the generation of mouse models with abrogated or enhanced Foxp3^+^ Treg cell activity.

### Lympho-Hematopoiesis in Mice With Constitutive Treg Cell Deficiency

Scurfy mice exhibit a spontaneous loss-of-function mutation in the gene encoding Foxp3, resulting in complete absence of Foxp3 protein and functional CD4^+^Foxp3^+^ Treg cells. In addition to systemic autoimmunity targeting several organs such as skin, lung, stomach and liver, severe lympho-hyperproliferation represents another hallmark of the scurfy phenotype, resulting in massively increased secondary lymphoid organs and peripheral immune effector compartments (82, 84).

In initial studies in 3 to 4-week-old scurfy mice, we unexpectedly noticed that the immature B cell compartment in the spleen was essentially completely absent, which appeared to be at odds with the existing mature B cell compartments at the same anatomical site. A more systematic analysis of peripheral B cell compartments revealed even higher numbers of mature B cells in lymph nodes of scurfy mice as compared to wildtype control mice (69). These observations raised several important questions, including the origin of mature B cells in scurfy mice and the possibility that scurfy mice exhibit a heretofore-unappreciated defect in B cell development in the BM, in addition to the well-known hematopoietic bias in scurfy mice towards macrophages (85, 86). When we extended our analysis to lympho-hematopoiesis, it became clear that adolescent scurfy mice exhibit severe B cell developmental defects with near-complete absence of B lymphopoiesis in the BM. Pre-B-II and immature B cell stages were found consistently below the detection level. Several lines of evidence such as the indistinguishability of the BM microenvironment of scurfy and Foxp3-proficient mice within the first week of life with regard to the capacity to support B cell development *in vivo* argue against a constitutive lympho-hematopoietic defect, but support the assumption of an ontogenetic acquisition of B cell developmental defects in scurfy mice. The apparent inconsistency between previously described significant residual B lymphopoietic activity in the BM of 28-day-old scurfy mice (39) and the almost entire block of B cell development observed in mice of the same age in our study is most likely due to differences in the kinetics of autoimmune manifestations, perhaps due to microbial flora variations between independent colonies of Foxp3-deficient mice.

Overall, our results were further corroborated by other recent studies showing that the constitutive absence of Foxp3^+^ Treg cells impinges on lympho-hematopoiesis in the BM although significant differences between those studies exist. In conjunction with aberrant B lymphopoiesis and in line with other reports, we detected aberrantly increased proportions and numbers of Lin^−^Sca-1^+^c-Kit^hi^ (LSK) cells early during ontogeny, suggesting that HSCs might be directly affected by inflammatory cytokines such as type I and type II IFNs. Analysis of *in vivo* reconstitution capacities of scurfy-derived HSCs in BM irradiation chimeras provided inconsistent results, with either substantially reduced or efficient although delayed reconstitution of lymphoid and myeloid lineages (38, 39, 87–89). Moreover, the underlying mechanism and in particular the direct and/or indirect roles of Treg cells remain a matter of debate. While it has been suggested that an altered cytokine milieu in scurfy mice promotes the differentiation of myeloid lineage cells at the expense of lymphopoiesis (87, 89, 90), indicating that the hematopoietic changes are not directly caused by the lack of Treg cells, additional mechanisms such as significantly increased cell death of developing B cells may affect B lymphopoiesis (88).

Nevertheless, our data indicate that the observed defects may not be exclusively mediated by systemic autoimmunity that negatively feeds back to BM hematopoiesis, as neonatal adoptive Treg cell therapy suppressed exacerbated production of inflammatory cytokines such as IL-17 and IL-6, but not IFN-γ and restored defective thymopoiesis, but was ineffective in recovering defective hematopoiesis and B lymphopoiesis. One possible explanation for this might be the failure to re-establish efficient production of CXCL12 and IL-7, which was consistently decreased in the BM of untreated scurfy mice (69). Thus, it appears likely that the aberrant B lymphopoiesis in scurfy mice is a consequence of the diversion of lymphoid progenitors from the B lymphoid lineage in favor of increased myelopoiesis (85–87, 89, 90) and the corporate action of the enhanced systemic production of helper T cell cytokines and locally reduced expression of B lymphopoiesis-promoting factors in the BM (69).

Overall, our observations indicate that re-establishment of immune homeostasis is not sufficient to reinstate hematopoietic homeostasis. We speculate that the adult BM niche might be irreversibly affected by constitutive Treg cell deficiency, which is in line with the severe bone loss afflicted with pronounced autoimmune pathologies, we and others (87) detected in scurfy mice.

### Lympho-Hematopoiesis in Mice With Acute Treg Cell Ablation

In contrast to the above described conventional mouse models and depletion strategies, the diphtheria toxin (DT)-mediated deletion of Foxp3^+^ Treg cells allows for efficient, selective and temporally controlled depletion of Foxp3^+^ Treg cells without affecting CD25^+^ effector T cells (91, 92) and thus has provided essential information on Foxp3^+^ Treg cell biology in health and disease.

However, striking differences exist with regard to depletion efficiency and severity of clinical symptoms after Treg cell ablation (93, 94). Two independent mouse models with an insertion of a DT receptor (DTR) into either the endogenous Foxp3 locus (91) or a Foxp3 promoter-encoding bacterial artificial chromosome [BAC; these mice were termed “depletion of regulatory T cells” (DEREG) mice, (92)] were generated in parallel. In both models, expression of the DTR under the control of the Foxp3 promoter and administration of DT leads to the induction of apoptotic cell death due to blockage of protein synthesis (95), specifically in Foxp3^+^ Treg cells. When DT was applied to neonatal mice, both, the BAC-transgenic and the knock-in approach resulted in an autoimmune disease similar in severity to that of Foxp3-deficient scurfy mice (91, 92). However, while DT-induced Treg cell ablation in adult mice did not induce scurfy-like disease in DEREG mice (92), Foxp3^DTR^ knock-in mice succumbed to a severe lympho-hyperproliferative disorder (91), which might be explained by different efficiencies with regard to transgene expression (93). Another study assessed BAC transgenic Foxp3DTR lines (Foxp3.LuciDTR, specifically generated for bioluminescence imaging of Treg cells) for depletion efficiencies and signs of pathology and demonstrated that lines with depletion efficiencies of >95% succumbed to a wasting disease, whereas lines with depleting efficiencies <95% lacked the onset of autoimmunity (96).

To deplete Foxp3^+^ Treg cells in adult steady-state mice, we therefore took advantage of the well-characterized DEREG mouse model (92–94, 97).

**Figure 2:**
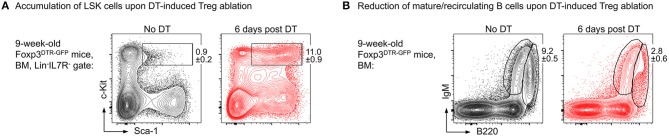
Impact of Foxp3^+^ Treg cell ablation on the BM niche. Foxp3^DTR−GFP^ mice were *i.p*. injected with DT on 3 consecutive days and the BM was analyzed at day 6 after the first DT dose by flow cytometry. Representative plots of **(A)** LSK (Lineage^−^IL7R^−^Sca-1^+^c-Kit^+^) cells and **(B)** mature/recirculating B cells (B220^high^IgM^+^) in the BM of 9-week-old untreated (left) and DT-treated (right) mice.

Our results show that upon administration of consecutive doses of DT, Foxp3^GFP+^ Treg cells in primary (thymus, BM) and secondary (spleen, lymph nodes, peritoneum) lymphoid organs of NOD.Foxp3^DTR−GFP^ mice were highly efficiently depleted with minimal systemic effects due to rapid recovery of the depleted Treg cell compartment. In contrast to DT-treated C57BL/6 and Balb/c mice, NOD control mice did not show any DT side effects. As a read-out for the impact of Treg cell activity on the generation of adaptive immune cells from HSCs, we assessed the developmental B lymphopoietic activity in the absence and presence of Foxp3^+^ Treg cells. Our comprehensive analysis of the BM niche revealed that acute Foxp3^+^ Treg cell ablation in Foxp3^DTR−GFP^ mice results in rapid dysregulation of lympho-hematopoiesis, as evidenced by a strongly increased HSC compartment size (Figure 2A) and a strong reduction of BM-residing mature B cells (Figure 2B). Of note, and in contrast to our results obtained in mice with constitutive Treg cell deficiency showing that B cell development was almost completely abrogated (69), we observed that acute Treg cell ablation in adolescent NOD.Foxp3^DTR−GFP^ mice induced only a slight reduction in B cell development. In contrast to scurfy mice, where already the earliest c-Kit^+^ Pro/Pre-B-I cell precursors were significantly reduced, this compartment remained unaffected by the DT-mediated Treg cell depletion, emphasizing that the effects observed in scurfy mice are not directly caused by Treg cell deficiency.

The apparent discrepancy between the recently reported almost complete block in B cell development observed in C57BL/6 Foxp3^DTR^ knock-in mice (98) and the only partial block in early B cell development we observed in NOD.Foxp3^DTR−GFP^ mice, could most likely be attributed to scurfy-like lethal autoimmunity and unwanted DT side effects that have been described for the former mouse model but not for the latter (91, 92, 94, 99, 100).

Overall, the prompt alteration of B lymphopoiesis in the BM upon DT-mediated Treg cell ablation in the absence of overt autoimmunity further support a potential direct role of Treg cells in hematopoiesis.

## Bone Homeostasis and Foxp3^+^ Treg Cells

### Osteoclast Development and Treg Cells

Osteoclasts, exclusive bone-resorbing cells of hematopoietic origin, are giant multinucleated cells derived from fused precursor cells of the myeloid lineage. The development of mature osteoclasts from early myeloid progenitors is a tightly controlled process involving multiple mediators within the bone microenvironment. Osteoclastogenesis is controlled by ligation of macrophage colony-stimulating factor (c-Fms; M-CSF) and membrane receptors for receptor activator of NF-kB ligand (RANK; RANKL). Under physiological conditions, osteoblast lineage cells, which are of mesenchymal origin, seem to be the main source for M-CSF and RANKL. The central role of M-CSF in survival and proliferation of osteoclast precursors was already revealed decades ago, by studying osteopetrotic op/op mice that bear a natural mutation in the *Csf1* gene coding for M-CSF (101, 102). The discovery of the RANK/RANKL/osteoprotegerin (OPG) signaling axis represents another milestone in bone biology: the finding that RANKL-deficient mice develop severe osteoclast-deficient osteopetrosis, whereas overexpression of RANKL or the lack of the decoy receptor for RANKL, OPG, result in osteoporosis, due to excessive osteoclast formation (103–105), emphasize the critical role of these pathways in osteoclastogenesis.

RANKL promotes the differentiation of committed osteoclast precursor cells by inducing the expression of several genes, such as *cathepsin K, tartrate-resistant acid phosphatase* (*TRAP*) and *calcitonin receptor*, as well as transcription factors, such as NF-κB, c-Fos, and the master transcription regulator of osteoclast differentiation NFATc1 (106). Important signals downstream of c-Fms include the PI3K/Akt pathway, crucial for cell survival and the MAPK/ERK pathway essential for survival and proliferation of precursor cells (4).

The discovery that activated T cells express RANKL along with the identification of RANKL as a key differentiation factor for osteoclasts represented one of the first links between the immune and the bone system (107–109). To date, the impact of numerous inflammatory cytokines on the fate of bone cells has been investigated *in vitro* and *in vivo*, and it has been shown that for example IL-1, TNF-α, IL-17, and IL-6 promote osteoclastogenesis by either inducing RANKL expression or directly acting on osteoclast precursor cells, whereas IL-10, IL-4, IFN-γ, and GM-CSF have inhibitory effects on osteoclast differentiation and function, illustrating the tight relationship of osteoclast precursor cells with other cells in the bone microenvironment such as innate and adaptive immune cells (9).

Despite extensive research efforts, osteoclast developmental stages as well as the precise identity of bona fide osteoclast precursors have remained poorly defined and thus there is still fundamental lack of reliable methods for *ex vivo* purification of osteoclasts and their precursors. For this reason, most studies are based on *in vitro* differentiated osteoclasts derived either from macrophages expanded *in vitro* in the presence of M-CSF or from unfractionated whole BM cells cultured under osteoclastogenic conditions, i.e., in the presence of M-CSF and RANKL. However, both methods have their drawbacks since interpretation of data obtained from these cultures is hampered by the heterogeneity of the starting population of which only a minor faction represents osteoclast progenitor cells that differ in their growth kinetics and differentiation potential. Moreover, isolation of primary cells from their *in vivo* microenvironment and subsequent *in vitro* culture results in altered phenotypic and functional characteristics of these cells.

To characterize and isolate osteoclast precursor cells, we combined complex state-of-the-art experimental approaches such as osteoclast differentiation and functional assessment (110, 111) with high-end flow cytometry and cell sorting of very rare cell populations employing a comprehensive array of hematopoietic cell surface markers suggested in varying combinations in the literature (112–116). Multicolor flow cytometry allowed for identification and prospective isolation of these infrequent cell subsets, which — in some cases — account for <0.1% of total BM cells in wildtype mice. To determine the osteoclastogenic potential of the identified populations, FACS-purified cells were cultured in the presence of M-CSF and RANKL. Differentiated osteoclasts were detected as giant, multinucleated, and TRAP-positive cells.

**Figure 3:**
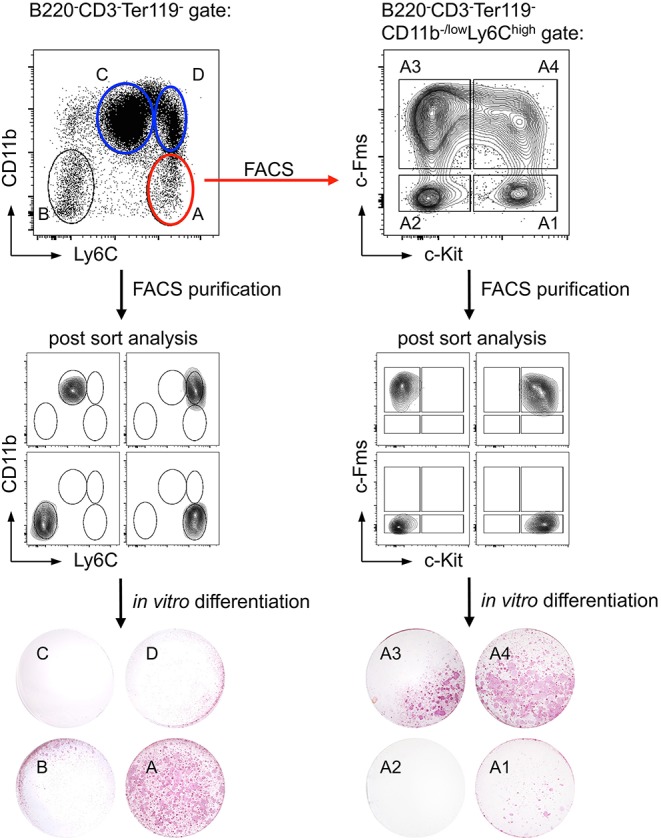
Identification of a cell population with high *in vitro* osteoclast differentiation potential. Ly6C and CD11b expression of bead-enriched B220^−^CD3^−^Ter119^−^ BM cells was detected by flow cytometry. Four distinct populations (A: Ly6C^high^CD11b^−/low^; B: Ly6C^−^CD11b^−^; C: Ly6C^low^CD11b^+^; D: Ly6C^+^CD11b^+^) were FACS-purified and after post-sort analysis *in vitro* differentiated under osteoclast-forming conditions (cytokines: M-CSF and RANKL). The differentiated cultures were fixed and a TRAP staining was performed to identify mature osteoclasts. Additionally, population A was further subdivided by the expression of c-Kit and c-Fms (A1: c-Kit^+^c-Fms^−^; A2: c-Kit^−^c-Fms^−^; A3: c-Kit^−^c-Fms^+^; A4: c-Kit^+^c-Fms^+^). After FACS-purification and post-sort analysis, the populations were cultured and analyzed for their osteoclastogenic potential.

With regard to CD11b expression on potential progenitor cells earlier studies have yielded contradictory results: while some groups described CD11b^+^ precursor cells to exhibit high osteoclastogenic potential, others reported on CD11b^−/low^ cells being more potent (114–118). In our hands, B220^−^CD3^−^Ter119^−^CD11b^+^ BM cells did not have a high osteoclastogenic potential, independent of the expression of Ly6C, whereas the B220^−^CD3^−^Ter119^−^CD11b^−/low^Ly6C^hi^ cell population contained the majority of osteoclast progenitors (Figure 3, left), which is in accordance with recent findings by Nakamura and colleagues (116). Since that study detected heterogeneous CD115 (c-Fms) and CD117 (c-Kit) expression on B220^−^CD3^−^Ter119^−^CD11b^−/low^Ly6C^hi^ osteoclast progenitor cells (116) and others identified CD115^+^CD117^+^ double positive cells as highly osteoclastogenic (113, 115), we applied in our study a combination of the surface markers used in these studies. Our results show that B220^−^CD3^−^Ter119^−^CD11b^−/low^Ly6C^hi^CD115^+^CD117^+^ BM cells efficiently differentiate into mature TRAP-expressing osteoclasts *in vitro* (Figure 3, right). Initial analyses of the BM niche of Foxp3^DTR−GFP^ mice provided first evidence that acute Foxp3^+^ Treg cell ablation results in substantial changes in osteoclast precursors in the BM.

The sorting of low-abundance prospective primary osteoclast precursors to high purity allow for detailed *ex vivo* analysis without contamination by unwanted cells and hence represents a powerful method to evaluate how Treg cells are affecting the formation of osteoclast precursors in mouse models with abrogated or enhanced Treg cell activity.

### Osteoclast Function and Treg Cells

Foxp3^+^ Treg cells control adaptive and innate immune responses by the suppression of activation, proliferation and function of various immune cell types, as for example CD4^+^ helper T cells, CD8^+^ cytotoxic T cells, B cells, NKT cells, macrophages, and dendritic cells (DCs). Multiple mechanisms of Foxp3^+^ Treg cell-mediated suppression have been proposed, involving cell contact-dependent and cell contact-independent mechanisms. The basic mechanisms used by Treg cells include (a) secretion of inhibitory cytokines, such as TGF-β, IL-10, and IL-35, (b) induction of cytolysis by for example granzymes, (c) metabolic disruption by e.g., IL-2-deprivation-mediated apoptosis, and (d) functional modification of antigen presenting cells (APCs) such as DCs. Expression of the cytotoxic T-lymphocyte antigen 4 (CTLA-4) enables Treg cells to interact with CD80/CD86 on DCs outcompeting CD28-mediated co-stimulation and thereby indirectly preventing differentiation of effector T cells (119).

In this context, it has been suggested that Treg cells have the ability to suppress osteoclastogenesis *in vitro*, but the mechanisms of suppression remain incompletely understood and controversial: while some studies identified inhibitory cytokines as key players in Treg cell-mediated suppression of osteoclasts, other reports suggested cell-cell-contact dependent mechanisms (40, 47, 48, 120–122). Most studies addressing the impact of Foxp3^+^ Treg cells on bone homeostasis *in vivo* employed mouse models with either constitutive Foxp3 deficiency or constitutive Foxp3 overexpression (48, 87, 123). As mice with constitutive Foxp3 deficiency suffer from a massive autoimmune lymphoproliferative disease and die approximately at 3 weeks of age (84) and overexpression of Foxp3 in CD4^+^ T cells *in vivo* results in reduced total numbers and functional impairment of T cells (124, 125) potential direct effects of gain and loss of Foxp3^+^ Treg cells are difficult to dissect from the impact of systemic immune dysregulation.

To our knowledge, the impact of temporally controlled, transient Foxp3^+^ Treg cell ablation (91, 92) on skeletal homeostasis under physiological conditions has not been addressed so far.

Osteoclasts and DCs share several features (126, 127). In this context, a recent study proposed that osteoclasts express the co-stimulatory molecules CD80 and CD86 and that Treg cells can regulate osteoclast differentiation via CTLA-4 (128). Moreover, it has been described that BM-residing Treg cells express higher levels of CTLA-4 than peripheral Treg cells (68). Therefore, we hypothesize that this direct cell-cell interaction may indeed play a central role in the crosstalk of osteoclasts and Treg cells.

**Figure 4:**
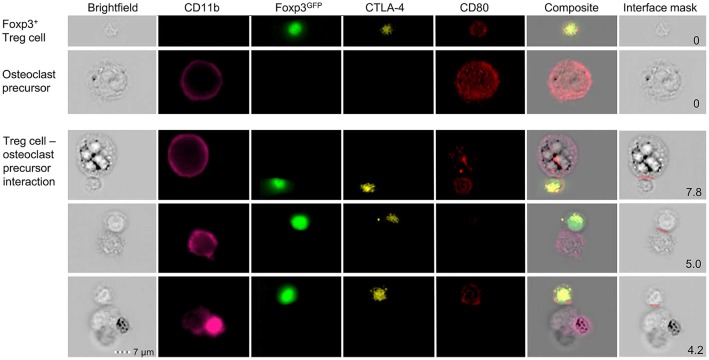
Detailed characterization of *in vitro* Foxp3^+^ Treg cell-osteoclast precursor interaction. Representative single cell (upper panels) and cluster (lower panels) analysis by imaging flow cytometry. Stimulated FACS-purified CD4^+^CD25^+^Foxp3^GFP+^ Treg cells and freshly isolated irradiated T-cell-depleted splenocytes were added to the BM culture and cultured under osteoclastogenic conditions. After 48 h, Foxp3^+^ Treg cells and CD11b^+^ osteoclast precursors were analyzed for intracellular expression of CD80 and CTLA-4 by imaging flow cytometry. Cell clusters depict physical interaction of CTLA-4^+^Foxp3^GFP+^ Treg cells and CD11b^+^ osteoclast precursors. Co-localization of CD80 in Treg cells indicates trans-endocytosis from CD11b^+^ osteoclast precursors. Numbers indicate the interface area and illustrate the overlapping fluorescence of Foxp3 and CD11b.

To investigate the direct interaction of osteoclasts and Treg cells, we performed *in vitro* co-cultures of Treg cells and BM-derived precursor cells. These experiments revealed that the expression of co-stimulatory molecules on the surface of osteoclasts is down-regulated in the presence of Treg cells which leads to the suppression of osteoclast differentiation and function. To directly study cell-cell interactions, cells harvested from these cultures were subjected to simultaneous analysis by multi-parameter flow cytometry and fluorescence microscopy. Imaging flow cytometry showed the direct cell-cell interaction between individual CD11b^+^ late osteoclast precursors and Foxp3^GFP+^ Treg cells which additionally exhibited intracellular red puncta (Figure 4), providing first direct evidence that Treg cells can remove CD80/CD86 from the surface of osteoclast precursors by CTLA-4 mediated trans-endocytosis, potentially leading to reduced co-stimulation by osteoclasts — a mechanism which has been described for the interaction of Treg cells and APCs such as DCs (129).

In sum, available data suggest that Treg cells can interfere with bone resorption by affecting osteoclast development and function at multiple stages. However, the mechanism of action is still controversial, emphasizing the complexity of this relationship.

Whether or not T cell regulation of bone and hematopoietic homeostasis requires APCs within the bone microenvironment, is still unknown, but based on the notion that osteoclasts are derived from hematopoietic precursors of the myeloid lineage, express a number of immune receptors and are regulated similarly to macrophages and DCs (130), it is a legitimate question, whether osteoclasts play a role in the active regulation of the immune system, can act e.g., as APCs. Indeed, albeit critically discussed (131), it has been suggested that, in addition to their ability to resorb bone, *in vitro* differentiated osteoclasts can function as APCs and activate CD4^+^ and CD8^+^ cells (132). In this context, it was reported that osteoclasts are capable to cross-present antigens to CD8^+^ T cells, thereby inducing Foxp3 in these cells, which were characterized as osteoclast-induced regulatory CD8^+^ T cells (133–135).

In addition, recent data suggest that osteoclasts can on the one hand participate in immunogenic T cell responses under chronic inflammatory diseases (136) and on the other hand exhibit immune suppressive functions in multiple myeloma (137, 138). Whether these proposed immune-modulating functions of osteoclasts play a role under physiological conditions or depend on the pathological conditions still needs to be defined. Data from our own group propose the concomitant up-regulation of c-Fms, RANK, and the ligand for the immune checkpoint molecule programmed death 1 protein (PD-1; PD-L1) on the surface of murine CD11b^+^ osteoclast precursors cultured under osteoclastogenic conditions, which complements the findings on human osteoclast precursor cells (137).

**Figure 5:**
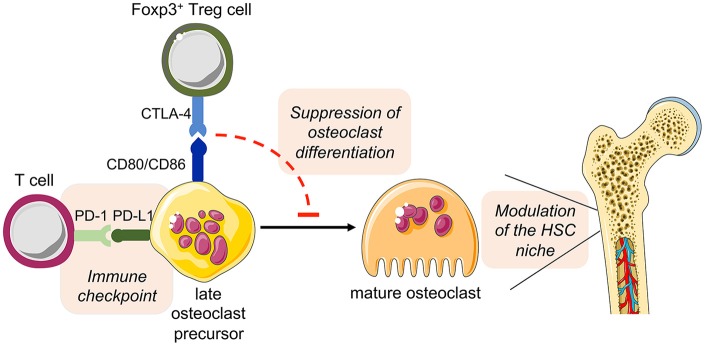
Hypothesized direct cross-talk between osteoclast precursors and T cells and the indirect effect on hematopoiesis. The expression of the costimulatory molecules CD80 and CD86 on osteoclast precursors has been implicated in the interplay with Foxp3^+^ Treg cells (via the inhibitory molecule CTLA-4), leading to the suppression of the differentiation of osteoclast precursors into mature functional osteoclasts. As a consequence, disturbed bone remodeling results in modulation of the HSC niche. On the other hand, osteoclast precursors are able to upregulate the expression of PD-L1, whose interaction with PD-1 on T cells has immune modulatory functions, thus representing a crucial immune checkpoint. This figure was in part created with modified Servier Medical Art templates, licensed under a Creative Commons Attribution 3.0 Unported license: http://smart.servier.com.

Overall, these observations open additional new prospects for the role of osteoclast in the intercellular interaction of bone cells and immune cells in health and disease (Figure 5).

## Conclusion

The clinical relevance of the BM niche is reflected by its function as (a) the primary hematopoietic site in the adult, (b) a reservoir for memory cells, and (c) in the support of HSC transplantation. The nature of the HSC niche in the BM is defined by various components of the BM microenvironment. Several independent lines of evidence are pointing towards specialized local functions of BM-residing Treg cells in settings of unwanted (autoimmunity, transplant rejection, GvHD) and insufficient immunity (cancer, chronic infections). Modulation of Treg cell activity *in vivo* can be achieved by means such as application of IL-2/anti-IL-2 immune complexes to enhance Treg cell function, whereas depletion or other means of interference with Treg cell function promotes immune responses. This raises the question whether comparable strategies may also be suitable to modulate bone remodeling to manipulate lympho-hematopoiesis in the adult BM. Are there niche-associated Treg cell subsets that specifically interact with bone cells such as osteoclasts? Overall, future studies are warranted to further define the molecular pathways involved in the complex intercellular communication between BM-residing Treg cells, hematopoietic stem and progenitor cells, mature immune cells, and the skeletal system. Understanding the immune regulatory mechanisms in the BM microenvironment is central for the development of effective therapeutic strategies for the treatment of bone diseases and to improve current protocols for HSC transplantation and peripheral immune reconstitution.

## Author Contributions

LF, CH, RK, SD, and JR designed, performed, and analyzed the experiments. KK and AG conceived the research, guided its design, analysis, and interpretation and wrote the manuscript. All authors contributed to discussions and writing of the manuscript.

### Conflict of Interest Statement

The authors declare that the research was conducted in the absence of any commercial or financial relationships that could be construed as a potential conflict of interest.
